# Influence of facial feedback during a cooperative human-robot task in schizophrenia

**DOI:** 10.1038/s41598-017-14773-3

**Published:** 2017-11-03

**Authors:** Laura Cohen, Mahdi Khoramshahi, Robin N. Salesse, Catherine Bortolon, Piotr Słowiński, Chao Zhai, Krasimira Tsaneva-Atanasova, Mario Di Bernardo, Delphine Capdevielle, Ludovic Marin, Richard C. Schmidt, Benoit G. Bardy, Aude Billard, Stéphane Raffard

**Affiliations:** 1 Learning Algorithms and Systems Laboratory, School of Engineering, EPFL, Lausanne, Switzerland; 2 EuroMov, Montpellier University, Montpellier, France; 3 University Department of Adult Psychiatry, CHU, Montpellier, France; 4 Department of Mathematics, College of Engineering, Mathematics and Physical Sciences, University of Exeter, Exeter, United Kingdom; 5 Department of Engineering Mathematics, University of Bristol, Bristol, United Kingdom; 6 Psychology Department, College of the Holy Cross, Worcester, MA USA; 7 Institut Universitaire de France, Paris, France; 8 Laboratory Epsylon, EA 4556, University Montpellier 3 Paul Valery, Montpellier, France

## Abstract

Rapid progress in the area of humanoid robots offers tremendous possibilities for investigating and improving social competences in people with social deficits, but remains yet unexplored in schizophrenia. In this study, we examined the influence of social feedbacks elicited by a humanoid robot on motor coordination during a human-robot interaction. Twenty-two schizophrenia patients and twenty-two matched healthy controls underwent a collaborative motor synchrony task with the iCub humanoid robot. Results revealed that positive social feedback had a facilitatory effect on motor coordination in the control participants compared to non-social positive feedback. This facilitatory effect was not present in schizophrenia patients, whose social-motor coordination was similarly impaired in social and non-social feedback conditions. Furthermore, patients’ cognitive flexibility impairment and antipsychotic dosing were negatively correlated with patients’ ability to synchronize hand movements with iCub. Overall, our findings reveal that patients have marked difficulties to exploit facial social cues elicited by a humanoid robot to modulate their motor coordination during human-robot interaction, partly accounted for by cognitive deficits and medication. This study opens new perspectives for comprehension of social deficits in this mental disorder.

## Introduction

Schizophrenia is characterized by symptoms such as delusions, disorganization and hallucinations. Social deficits are as well a core feature of this disorder^1,2^. In particular, the impoverished ability to process social information and to modulate interpersonal behavior accordingly have severe negative impact on schizophrenia patients’ social life^3,4^. Engaging and maintaining social interactions relies to a large extent on non-verbal cues. An abundant literature demonstrates that patients with schizophrenia perform poorly when requested to interpret cues conveyed by facial emotions^5^, hand gestures^6^, body postures^7^ and gaze direction^8^. Among these, facial emotion interpretation has been reported to be highly associated with social competence in schizophrenia^9,10^. Non-verbal cues, such as facial emotions, provide feedback about intentions and emotional states of others, that influences our behavior^11–14^. The ability with which two partners coordinate their movements with one another is often quantified through a measure of synchrony across the two partners’ movements^15–17^. It has been shown that the affiliation between co-actors can lead to spontaneous synchronization of body movements^18^. Conversely, synchrony during social interaction can lead to a higher affiliation rating and cooperation between individuals^19^. Recent studies suggest a general impairment in interpersonal synchronization in patients with schizophrenia^20,21^. However, those studies do not explore the specific influence of social feedback on interpersonal synchronization. This constitutes the core question of our work. In particular, we hypothesize that non-verbal social cues influence our ability to coordinate our movements with that of others, even in very simple motor imitation tasks. We refer to this interplay between social cues and motor synchrony as *social motor coordination* ^22^. This study is of particular relevance in the context of schizophrenia that affects both synchrony and the interpretation of social cues. To validate our hypothesis, traditional human-human interaction studies of social-motor coordination are limited due to the impossibility to precisely controlling for the social feedback. Therefore, one cannot separate easily the effect of the type (social versus nonsocial) and the frequency of feedbacks on the interaction. To be able to explore quantitatively the link between social feedback and motor synchrony, it is critical to provide comparable and controlled social cues during the interaction.

Socially assistive robotics (SAR) stem from a trend that endows robots with social, emotional and cognitive competences to enhance human-robot interactions. SAR have been used to assess social competences and therapeutic treatment for medical conditions with deficits in social competences, such as dementia^23^ or autism^24–26^. There is a wealth of applications of SAR as a diagnosis tool to provide a systematic assessment of symptoms related to social deficiencies in children with autism spectrum disorders (ASD)^27–29^. Moreover, SAR can improve engagement and elicit novel social behaviors in ASD individuals, including in subjects who do not interact socially with human therapists^30,31^. This line of research develops simple social interaction tasks in order to motivate and engage patients to interact socially with the robot. Social skills learned during these human-robot interactive sessions can then be transferred to similar interactions with human partners^32^.

As schizophrenia shares social symptomatology with ASD, such as a social withdrawal, an impoverished theory of mind and impairments in the interpretation of facial social cues, we posit that the promising results of SAR for ASD could be extended to schizophrenia. Only one study has used pet robots in schizophrenia, aiming at promoting social and emotional functioning in institutionalized patients^33^. However, to our knowledge, human-robot interaction during a collaborative task has never been exploited to monitor and study social interactions in schizophrenia.

In this work, we propose to use iCub, a humanoid robot able to display controlled social feedback in the form of facial smiles. Our study is hence the first attempt at assessing the potential of humanoid robots to study social cognition in schizophrenia. We selected a simple mirroring task, which consists for the subject in following the robot’s hand motion as accurately as possible. In our approach, the robot is endowed with the ability to adapt the amount and type (i.e., social or nonsocial) of feedback it gives to its human partner based on their synchrony: the more synchrony, the more positive feedbacks. As synchrony can induce an increase in affiliation, this, in turn, leads to an increase in the dyad’s motor coordination (i.e., affiliation-induced synchrony). The difficulty is in solving the causal ambiguity of this interactive loop (i.e., moving from correlation to causation). To help resolve this problem, we introduce a third condition in which the robot offers a neutral face. This serves as a baseline to break one link of the loop. We evaluate the effect of the type of feedback (i.e., social and nonsocial) provided and the frequency at which the feedback is generated, in three conditions:

1. a neutral condition where no feedback is displayed
2. a nonsocial condition, where a tablet mounted on the robot’s head displays a plus sign, and
3. a social condition where the robot displays a smiling facial expression using luminous color light-emitting diodes under the surface of its face, representing the mouth and the eyebrows.

The nonsocial and the social feedbacks are triggered in real time during the interaction by the same algorithm, according to the participant’s performance. In both cases, the feedback is displayed for one second, and is followed by a refractory period of 3 seconds were nothing is displayed (i.e., a neutral face for the social condition, and the tablet without the cross in the nonsocial condition). To obtain congruent feedbacks across conditions, we ensured that the surface covered by the luminous mouth and eyebrows of the iCub robot is the same as the surface covered by the cross on the tablet. Furthermore, the color used to trace the cross is the same as iCub’s facial diodes. Finally, the luminance is equivalent as both feedbacks are displayed by light-emitting sources on a white background.

**Figure 1:**
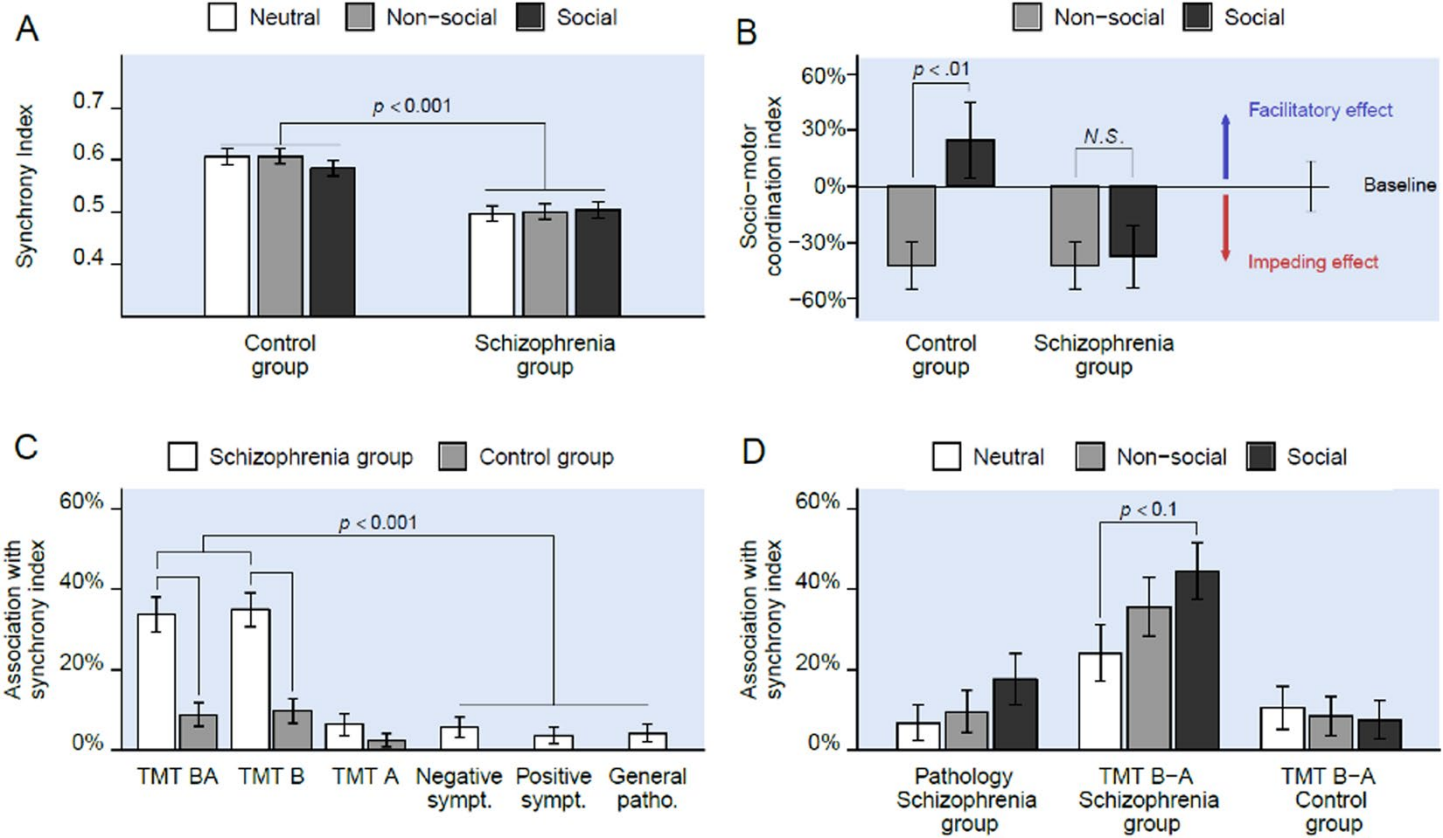
Overall results of the statistical analysis. (**A**) The schizophrenia group, compared to the control group, has a lower measure of synchrony regardless of the existence and the type of the feedback. (**B**) For the control group, the social feedback has a facilitatory effect on the motor coordination. In contrast, for the schizophrenia group, the social feedback has an impeding effect on the motor coordination. (**C**) For patients with schizophrenia, the synchrony index during the interaction is associated (i.e., negatively correlated) with cognitive abilities more than with symptomatology. (**D**) The association (i.e., the negative correlation) between cognitive flexibility and synchrony index is stronger in the presence of the social feedback. Such an observation is not present for the control group.

Furthermore, we explored clinical correlates using clinical standardized evaluation of symptoms severity in schizophrenia. Besides the clinical assessments, all participants were evaluated on the Trail Making Test (TMT-A and B) for cognitive functioning. We also characterized the participants with a measurement of Theory of Mind using the Mind Perception Questionnaire (MPQ) after the end of the trials. The MPQ is designed to evaluate how individuals perceive living and non-living things in terms of *experience* (e.g. How much is the robot capable of experiencing physical or emotional pleasure?) and *agency* (e.g. How much is the robot capable of thinking?). This setting is illustrated on Fig. 1. We exploit this setting to validate three hypothesis. First, we hypothesize that the general impairment in synchrony of patients with schizophrenia transfers to the interaction with a humanoid robot. Second, we hypothesize that positive social feedbacks should facilitate interpersonal synchronization in control subjects. However, we expect that this facilitation effect is not present in schizophrenia patients, due to their impairment in dealing with social cues.

## Results

### Participants Group Comparisons

With respect to age and gender, healthy control subjects were matched to patients with schizophrenia; see Table 1. The median age of the patients (Mdn = 29) and the healthy control subjects (Mdn = 28) were statistically comparable (U = 218, z = −0.56, p = 0.57, r = 0.09). The ratio of male to female participants in each group (17/5 for patients, and 15/7 for control group) did not differ significantly (χ2(1) = 0.11, p < 0.73).

**Table 1:** Demographic characteristics of the participants. Median and range [minimum-Maximum] for non-parametric tests; Education: years of education; NSS: Neurological soft sign scale; TMT: Trail Making test; PANSS: positive and negative syndrome scale.

	Schizophrenia participants (n = 22)	Matched controls (n = 22)	Statistics	Sig.
Age (years)	29 [21–45]	28 [19–46]	U = 218^a^	0.58
Sex (male/female)	17/5	15/7	X^2^ = 0.11^b^	0.73
Education (years)	12 [9–17]	12 [9–17]	U = 240^a^	0.97
TMT-A (seconds)	28.5 [17–57]	21 [15–38]	U = 110^a^	0.002
TMT-B (seconds)	75.5 [35–150]	47 [32–180]	U = 125^a^	0.006
TMT-(B-A) (seconds)	42.5 [15–116]	26 [12–156]	U = 148^a^	0.028
NSS	19.2 [6–38.5]	16.3 [1.5–22.3]	U = 125^a^	0.006
PANSS Positive	9.5 [7–18]			
PANSS Negative	10 [7–22]			
PANSS Psychopathology	22 [17–35]			
PANSS Total	43 [31–66]			

^a^Mann-Whitney test.

^b^Chi-squared test.

### Synchrony & Social-Motor Coordination

To study the effect of the nature and frequency of feedback on the motor coordination, a multiple linear regression was performed. This model predicts the synchrony index based on the group, the nature of the feedback and its frequency. A significant regression equation was found (*F*(9, 649) = 23.2, *p* < 0.001), with *R* ^2^ = 0.24; see supplementary materials. This model showed that the schizophrenia group has, irrespectively of the nature and the frequency of the feedback, a lower synchrony with the robot during the coordination task compared to the control group (*F*(1, 649) = 25.06, *p* < 0.001); see 1A.

Moreover, the linear regression showed the sensitivity of synchrony to the frequency of the feedback in each condition (*SMCi*). This metric was first exploited to study the effect of the presence of any type of feedback (social and nonsocial), averaged over both control and schizophrenia groups. The results showed a decrease of the *SMCi* of 42% in presence of feedback compared to the neutral case (*F*(1, 649) = 5.196, *p* = 0.023). Secondly, the *SMCi* was used to contrast the influence of social feedback compared to nonsocial feedback. The social feedback condition improved the *SMCi* by 85% (*F*(1, 649) = 3.884, *p* = 0.049). Furthermore, this effect interacted with the group. The social feedback resulted in a higher *SMCi* in the control group compared to the schizophrenia group (*F*(1, 649) = 5.607, *p* = 0.018). The normalized measure of *SMCi* across conditions is illustrated in Fig. 1B. As can be seen, with respect to *SMCi*, the only statistically significant difference across group lied in the social condition.

### Examination of Confounding Factors

We conducted a second linear regression, controlling for variables for which the groups were matched; i.e., age and gender. Although no statistically significant effect of age was detected (*F*(1, 647) = 1.129, *p* = 0.288), this model showed that female participants, compared to male participants, had a lower measure of synchrony (*F*(1, 647) = 50.0167, *p* < 0.001). No statistically significant effect of the different robot hand’s trajectories was detected (*F*(4, 654) = 0.534, *p* < 0.711). The results obtained in terms of synchrony and *SMCi* according to these observations remains consistent with our analysis.

### Clinical Correlates

To gain insight into the pathological underpinning of schizophrenia patients’ impairment in this social coordination task, we conducted a correlation analysis between synchrony index and clinical evaluation of symptom severity in the patients group. Our findings showed that patients’ performance regarding synchrony was highly negatively correlated to cognitive flexibility as indexed by the difference between TMT B and TMT A (TMT B-A) performances (*R* ^2^ = −0.34, *p* < 0.001). This correlation was statistically stronger not only compared to psychotic symptomatology (*z* > 5.23, *p* < 0.001), but also compared to the correlations found in the control group (*z* > 4.48, *p* < 0.001). These correlations and their significant pairwise comparisons are illustrated on Fig. 1C.

Furthermore, we explored the correlations between measures of clinical evaluation and the synchrony index across conditions; see Fig. 1D. These correlations suggest that in schizophrenia patients, the synchrony index was negatively correlated to TMT B-A performance more than to the combination of psychotic symptoms, especially in both social and nonsocial feedback conditions (*z* > 2.55, *p* < 0.011). Focusing on the correlation between the synchrony index and TMT B-A performance for patients with schizophrenia, an increasing trend was observed across conditions. However, only the difference between neutral and social conditions was statistically marginally significant (*z* = 1.91, *p* = 0.055). Finally, the synchrony index was highly correlated with NSS for both patients with schizophrenia (τ = −0.15, *p* = 0.000) and control participants (*τ* = −0.26, *p* = 0.000).

The statistical analysis of the Mind Perception Questionnaire did not show any group (patients or controls)-dependent effect of agency attribution on the performance in terms of synchrony. Detailed statistical analysis and results are given in the Supplementary Materials.

Finally, we explored the correlation between the medication dosage (chlorpromazine equivalents CPZ) and the synchrony index. The synchrony index is negatively correlated to the CPZ in general (τ = −0.10, *p* = 0.01). Focusing on the different conditions, we observe a statistically significant correlation in the facial condition (τ = −0.13, *p* = 0.05), but no statistically significant correlation in the tablet condition (τ = −0.07, *p* = *0*.31) and the neutral condition (τ = −0.11, *p* = *0*.14).

## Discussion

In this study, we explored the effect of social feedback on motor coordination in schizophrenia during a collaborative task with a humanoid robot. Our objectives were: first, to explore if interpersonal motor coordination impairments already shown during interactions with a human partner^20,21^ also apply to interactions with a robot partner; second, to investigate social-motor coordination in schizophrenia by quantifying the effect of social feedback on motor coordination; and third, to clarify the factors underlying abnormal behaviors observed during the cooperation task.

First, we confirmed that patients with schizophrenia are impaired in their ability to synchronize with a robot partner in simple motor imitative tasks compared to control subjects. This extends previous findings of synchrony impairments in schizophrenia in human-human interactions^20,21^ to the interaction with humanoid robots.

More specifically, we investigated how social feedbacks influences the synchrony of the participants during the interaction. We showed that, compared to the neutral condition, the nonsocial feedback deteriorated motor synchrony to the same extent for both control and schizophrenia groups. We speculate that even though the nonsocial feedback is task-relevant (i.e., computed based on the quality of the interaction), its relevance to the task remained unclear to both control and patients with schizophrenia. Therefore, participants were unable to exploit nonsocial feedback as a cue to improve synchrony. Moreover, it may have shifted the participants’ attention away from the robot motions, thus deteriorating the synchrony. This hypothesis is supported by studies showing that visual attention modulates the strength of interpersonal coordination^34^.

Second, our findings showed that only nonclinical participants benefited from the social feedback elicited by the robot and that this feedback modified their movement accordingly to the proposed coordination goal. To our knowledge, this study is the first to demonstrate that social feedback improves coordination behavior more than nonsocial feedback in control subjects. Comparing the results obtained with social versus nonsocial feedback shows that the emotional content of the feedback facilitated the interaction in control subjects and not the task-related aspects of the feedback.

Importantly, our results revealed that compared to controls, patients with schizophrenia failed to improve their social-motor coordination in the presence of social feedback. This absence of facilitation in schizophrenia may be due to an impairment in the automatic link between perception of social cues (i.e., positive facial emotions such as smiles) and motor coordination. This hypothesis is in line with a body of literature that investigates the impairments in automatic processing in schizophrenia^35–39^. Alternatively, the lack of a facilitatory effect of social cues in schizophrenia participants may be due to specific cognitive deficits, such as an impaired ability to perceive and interpret facial social cues (i.e., smiles). In a preliminary study, we showed that patients with schizophrenia were able to accurately recognize the valence of facial emotion elicited by the same iCub robot^40^. This speaks against the concern expressed above.

Finally, our results showed a high negative correlation between patients’ social-motor coordination and their performance in the Trail Making Test (TMT). This correlation was stronger than the correlation with positive, negative symptoms and other clinical symptoms. The TMT B-A score has been shown to be a valid measure of cognitive flexibility, one of the main dimensions of executive functioning^41^. Cognitive flexibility is the ability to shift from one cognitive operation to another one^42^ and to interrupt automatic responses to come back to top-down cognitive control^43^. In non-pathological individuals, the motor-coordination response to social feedback is automatic, and thus does not rely on top-down cognitive control. Our results are thus in line with this observation, as the correlation between synchrony and TMT results was low for control participants.

For schizophrenia participants, a high correlation was obtained, particularly concerning the part B of the TMT; the part B of the TMT is considered as a test of higher level cognitive abilities such as mental flexibility. This finding supports the hypothesis that impairments in the brain circuits related to social processing are compensated by higher cognitive processes such as those involved in cognitive flexibility^44,45^. In the context of such an impairment, coordination of rhythmic behavior between individuals with schizophrenia engaged in a joint activity is a demanding task as it requires both precision and flexibility^46^. Our cooperation task is particularly demanding in that the participant has to simultaneously take into account the robot’s facial feedback and coordinate his/her movements with those of the robot while concurrently monitoring the overall integrated ensemble output. Patients with schizophrenia may fail to coordinate their own actions with others’ actions while maintaining effortful control of their own movements. There is abundant evidence that patients with schizophrenia have difficulty using positive feedback to adaptively guide their behavior^47,48^.

Furthermore, we observed a significant correlation between the synchrony index and medication dosage for the patients. This shows that patients treated with high dosage do not benefit from the facial cues as well as patients with lower dosage. In^49^, authors analyzed the effect of chlorpromazine equivalents on facial emotion perception. Their results suggest a marginal relationship between higher dosage and greater degree of impairment on tests of facial emotion perception. This observation could explain our result.

Our study has some limitations. First, our sample size is relatively small. Therefore, the results should only be generalized with caution. A second limit concerns the social cues used (i.e. facial feedback). The facial expressions of the robot include only a very narrow aspect of the complexity of a real human facial expression. Future work should explore more realistic and rich social cues, such as gaze, spoken language or haptic communication for the study of physical interactions between humanoids robots and individuals with schizophrenia. Furthermore, the processes by which patients in an interaction start, maintain and end their perceived connection to a robot needs to be further explored. Another important limit of our study concerns the lack of a visual perception measure. Indeed, patients with schizophrenia are known to be impaired at organizing and exploring the visual environment^50^ which can affect visual organization in space, the processing of low-spatial frequencies, and the pattern of eye movements. Further studies using for example eye-tracking methodology to assess participants’ gaze toward the robot during the task are thus needed.

To our knowledge, this is the first study that examined human-robot interaction in the context of a cooperation task in individuals with schizophrenia. Unlike with robots, one cannot easily manipulate how often and in which manner humans express social cues. This study exploited the fact robots can be used to provide social cues in a controlled way. Specifically, it offered a first systematic assessment of the effect of providing social positive feedback on schizophrenia.

In our study, patients with schizophrenia displayed reduced cooperation ability compared to controls during human-robot interaction through all conditions and, in particular, in the social cue condition. This may be due to the patients’ impoverished ability to process the social cues expressed by the humanoid robot or to their general inability to use social cues to modulate their behavior. In addition, we observed that antipsychotic medication affected the patients’ performance negatively. This suggests that antipsychotic medication reduces patients’ social competences. This result is in line with other studies showing an impeding effect of neuroleptics on emotional facial expressions recognition in schizophrenia spectrum disorders^49^.

Our study evaluated only one type of social feedback, namely positive feedback conveyed through a smiling face. Social feedbacks are crucial to successful interaction and communication and are conveyed by different modalities. Further studies are needed for evaluating and comparing the effectiveness of other types of feedbacks (e.g. verbal or haptic) in schizophrenia. Social robots, and in particular humanoid robot, may offer a useful tool, in this endeavor, as one can manipulate also their gaze and haptic interactions.

All patients accepted easily to interact with the iCub robot and engaged naturally in the interaction with the robot. This provides positive evidence of the acceptability of humanoid robots for further interaction protocols with schizophrenia patients. The rapid progress in humanoid robotics offers tremendous possibilities for innovation in the study of social interaction deficits. All the above lead us to conclude that robots constitute promising tools for studying social dysfunctions in patients with schizophrenia.

Our study was motivated by the wealth of publications showing the potential of social robots to accompany rehabilitation protocols in ASD, a mental disease bearing similar deficits in social cognition to schizophrenia. However, unlike patients with ASD who are responsive to simple social features emitted by robots, patients with schizophrenia did not benefit from the robot’s social feedback. We cannot exclude that this may result from the simplicity of the social cues generated by the robot. If one had used social robots bearing a stronger resemblance to human faces, such as androids^51^, the effect may have been different. Further studies using alternative types of social robots may help to confirm or infirm the potential of robots as a tool for therapeutic enhancement of social abilities in schizophrenia. Our study assessed the effect of social cues on a single session with patients who had no previous experience interacting with the robot. As therapeutic effects can only be assessed through repeated sessions, further works should explore the effect of interaction sessions with the robot on the long term.

## Methods

### Participants

#### Participants and exclusion criteria

We recruited 44 participants; 22 schizophrenia outpatients, and 22 age and gender-matched healthy participants. Patients were recruited from the University Department of Adult Psychiatry (CHRU Montpellier, France) and fulfilled the Diagnostic and Statistical Manual of Mental Disorders criteria for schizophrenia.

The control participants were recruited in the Montpellier area. They were screened for current psychiatric illness using the Mini-international Neuropsychiatric interview. The control participants did not meet any criteria for current axis I disorder of the DSM-IV-TR.

Exclusion criteria for both the clinical and nonclinical groups were (a) history of head trauma, (b) known neurological disease, (c) an actual ECT treatment, (c) substance abuse and or substance dependence (excluding tobacco and cannabis), and (d) people deprived of their liberty. All participants were native French speakers with a minimal reading level (validated using the fNART test) and were able to understand and perform the social-coordination task described in the following section.

All patients were taking medication, and doses were converted in chlorpromazine equivalents (mean dose = 286 mg, *SD* = 118, see Table 2). Patients received a neuroleptic treatment, either typical (*N* = 1) or atypical (*N* = 21). One patient was also administered with an antiparkinsonian treatment. Six patients were treated with benzodiazepines.

**Table 2:** Medication of the participants with schizophrenia.

Patient Number	Chlorpromazine equivalents (mg.)
1	400
2	125
3	400
4	200
5	200
6	200
7	135
8	400
9	400
10	135
11	200
12	200
13	400
14	400
15	250
16	450
17	200
18	250
19	400
20	250
21	500
22	200
Mean	286
SD	118

All patients were interviewed by members of the specialized multidisciplinary team of the University Department of Adult Psychiatry, which belongs to a French national network of 10 Schizophrenia Expert Centers (Bordeaux, Clermont-Ferrand, Colombes, Créteil, Grenoble, Lyon, Marseille, Montpellier, Strasbourg, Versailles), set up by a French scientific cooperation foundation, FondaMental Foundation (www.fondation-fondamental.org) and created by the French Ministry of Research.

Except for the SCID, patients were assessed by trained clinical psychologists who rated the PANSS and other clinical scales after a unique clinical interview.

All participants provided written informed consent, prior to the experiment approved by the National Ethics Committee (CPP Sud-Méditerranée-III, Nîmes, France, #2009.07.03ter and ID-RCB-2009-A00513-54) and conforming to the Declaration of Helsinki. Participants were evaluated on the Neurological Soft Signs Scale (NSS)^52^ to assess subtle abnormalities in sensory-perceptual motor functions directly associated with schizophrenia^53,54^ or induced by neuroleptic medications^55^. Patients also completed the Positive and Negative Syndrome Scale (PANSS)^56^. Cognitive assessment included part A and B of the trail making test (TMT). The difference between the completion time of TMT A and TMT B was used to provide an indicator of cognitive flexibility^57^. See Table 1 and supplementary materials for further details.

### Design

#### Human-robot collaboration task

**Figure 2:**
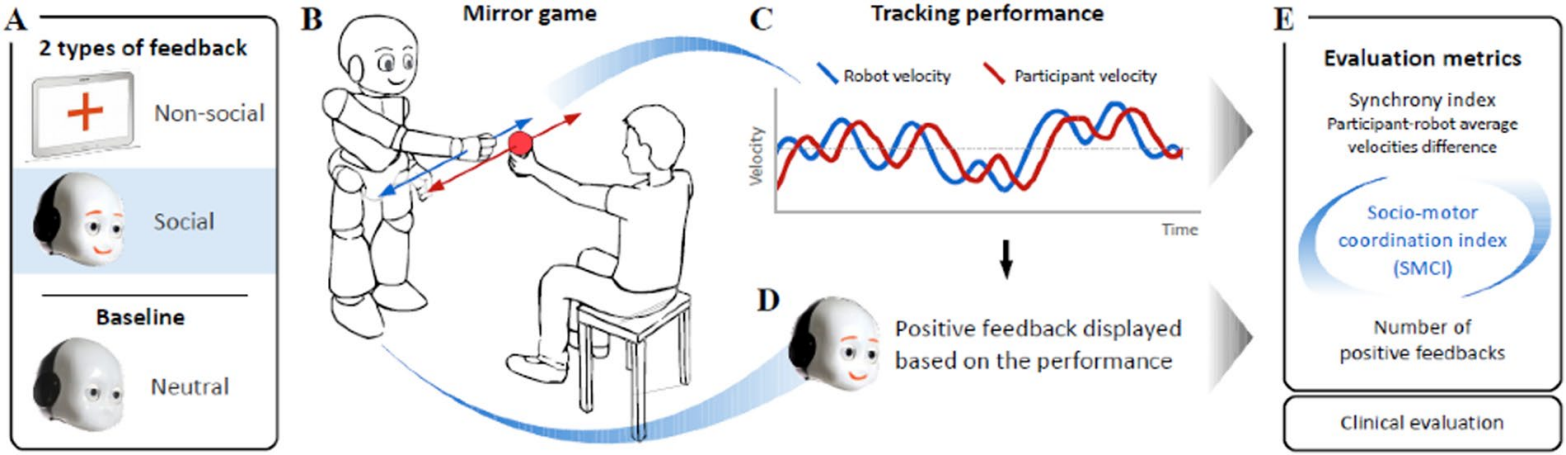
Schematic of the protocol. (**A**) Types of feedback displayed by the robot. (**B**) Human-robot collaboration task. (**C**) Evaluation of the quality of the interaction based on synchrony. (**D**) Feedback is displayed when the synchrony index increases. (**E**) The socio-motor coordination index measures the sensitivity of the synchrony to the frequency of the feedback.

We used the iCub^58^, a 1.20 m tall humanoid robot with 53 degrees of freedom, designed to offer a platform for the study of cognition and for human-robot social interactions. The quality of the collaboration between the robot and the participant was evaluated in the context of an imitation task called the mirror game^59^, whereby two players mirror each other’s hand motions. The robot provided the user with positive feedback whenever the synchrony between their motions improved. Feedback was either social or nonsocial (see Fig. 2). The interaction was hence composed of two aspects: motor coordination through the imitation game, and social non-verbal communication through the robot’s feedback. This socio-motor coupling enables a collaboration between the robot and the participant.

Participants were instructed to follow the hand movements of the robot with their own hand. The robot was programmed to play the mirror game as an assistive leader^60^. This enabled the robot to adapt to slowdowns in the case that the participant was lagging importantly. This assistive mechanism ensures to maintain the interaction even with poorly synchronized participants. To provide diversity in the robot’s movement, the robot switched across five different reference trajectories. To assure that the robot’s motion was human-like, the trajectories were generated according to a human-movement framework presented in past research^61^. The behavior of the robot (i.e., control parameters) was fixed throughout the experiment, ensuring that we observe only the effect of manipulated variables, i.e., nonsocial and social feedback.

**Figure 3:**
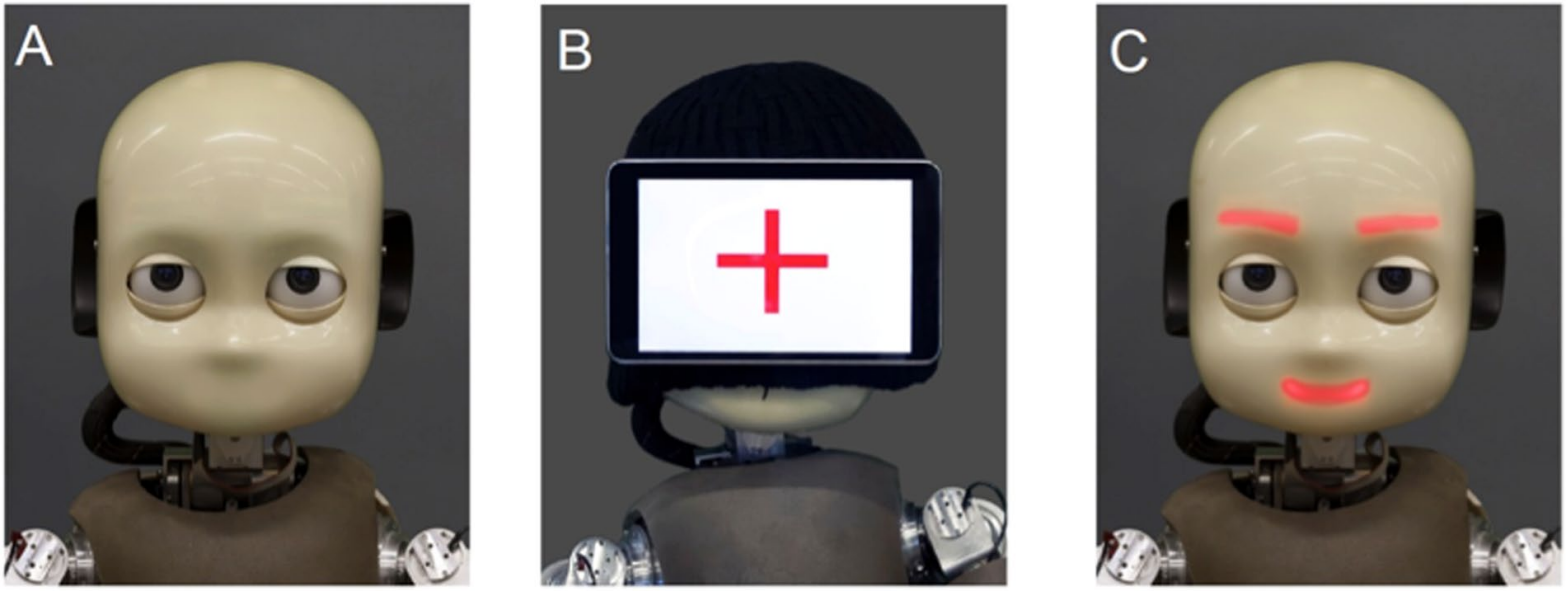
The three conditions of the experiment. (**A**) Neutral face (**B**) Nonsocial positive feedback (**C**) Social positive feedback.

The participant sat in front of the robot and engaged in the coordination task with all possible combinations between conditions (i.e., neutral, nonsocial, and social feedback) and robotic leading behaviors (i.e., 5 different motion signatures); see Fig. 3. This means that each participant performed 15 randomly-ordered trials, each trial lasting 60 seconds. In order to record the participants’ motions, they were asked to hold a red ball attached to a handle that was tracked by a camera mounted on the ceiling. This led to total of 660 recorded trajectories for the analysis.

#### Positive social and non-social feedback

The three conditions used are contrasted in Figs 2A and 3: in the social condition, the robot generated a smile using its facial LEDs; in the nonsocial condition, a plus sign was displayed on a tablet fixed on the robot’s head, hiding its face; in the baseline condition, the robot had a neutral face. Feedback was triggered each time the coordination was improved with respect to (1) position error, (2) velocity error, and (3) sum of velocities, compared to the last 5 seconds. Even though no positive feedback was displayed in the baseline condition, we still computed the number of events that could trigger the feedback; see supplementary materials for further details. This served to contrast participants’ synchrony across conditions. Participants took part in all three conditions in randomized order.

### Statistical analysis

The experimental design was composed of one independent between-subject variables, a group factor (control and schizophrenia), and one independent within-subject variable, a condition factor (neutral, nonsocial, and social). The number of positive feedbacks during the interaction was considered as a covariate. Demographic characteristics were statistically compared across groups using non-parametric U-Mann-Whitney tests for continuous variables (e.g., age), and Chi-squared tests for binary variables (e.g., gender). Pairwise comparisons between groups were performed using t-tests when necessary.

To evaluate synchrony, we computed the average velocity error between the participants and the robot. This measure (i.e., Synchrony index) is used as the dependent variable in our statistical analysis to study the effect of group and condition. A dummy variable (i.e., Feedback) compares the social and nonsocial conditions with the neutral condition; and a nested dummy variable (i.e., Social) compares the social to the nonsocial condition. To study the sensitivity of the synchrony index to the number of positive feedbacks, the frequency of the feedback was included in the model as a covariate. The estimated slope for this covariate shows how the synchrony index and the frequency of feedback are correlated (i.e., Socio-Motor Coordination index or *SMCi*). The estimated slope in the neutral case was used as a baseline.

For the clinical correlation analysis, we used a linear regression with the synchrony index as the dependent variable. To compare the correlation coefficients, we used a two-tailed Fisher *z*-score test.

We explored the correlation between medication dosage and synchrony with a Kendall Tau test.

### Data and materials availability

Fully anonymised data is available upon request.

## Electronic supplementary material

Supplementary Materials
