# Supplementary material for: Influence of facial feedback during a cooperative human-robot task in schizophrenia
Source: Sci Rep. 2017 Nov 3;7:15023. doi: 10.1038/s41598-017-14773-3 (PMC5670132; doi:10.1038/s41598-017-14773-3)
Supplement: Supplementary file 1 — Supplementary Materials [file 41598_2017_14773_MOESM1_ESM.doc]

**Supplementary Materials**

**Influence of facial feedback during a cooperative human-robot task in schizophrenia**

Laura Cohen^1,+^, Mahdi Khoramshahi^1,+^, Robin N. Salesse^2^, Catherine Bortolon^3^, Piotr Słowin´ski^4^, Chao Zhai^5^, Krasimira Tsaneva-Atanasova^4^, Mario Di Bernardo^5^, Delphine Capdevielle^3^, Ludovic Marin^2^, Richard C. Schmidt^6^, Benoit G. Bardy^2,7^, Aude Billard^1^, and Ste´phane Raffard^3,*^

^1^Learning Algorithms and Systems Laboratory, School of Engineering, EPFL, Lausanne, Switzerland

^2^EuroMov, Montpellier University, Montpellier, France

^3^Laboratory Epsylon, University Department of Adult Psychiatry, CHRU, Montpellier, France

^4^Department of Mathematics, College of Engineering, Mathematics and Physical Sciences, University of Exeter, United Kingdom

^5^Department of Engineering Mathematics, University of Bristol, United Kingdom

^6^Psychology Department, College of the Holy Cross, Worcester, MA, USA

^7^Institut Universitaire de France, Paris, France

^+^These authors contributed equally to this work

**Participants and exclusion criteria**

We recruited 44 participants; 22 schizophrenia outpatients, and 22 age and gender-matched healthy participants. Patients were recruited from the University Department of Adult Psychiatry (CHRU Montpellier, France) and fulfilled the Diagnostic and Statistical Manual of Mental Disorders criteria for schizophrenia. All patients were interviewed by a trained psychiatrist with the Structured Clinical Interview for Mental Disorders (SCID 1.0) to confirm the diagnosis. All patients received antipsychotic medication.

Age and gender-matched healthy participants were recruited from a call for participation on the hospital’s website and local community. They had no lifetime history of any psychosis diagnoses according to the SCID. The healthy subjects were matched by age, sex and education level with schizophrenia patients. The control participants were recruited in the Montpellier area. They were screened for current psychiatric illness using the Mini-international Neuropsychiatric interview. The control participants did not meet any criteria for current axis I disorder of the DSM-IV-TR. All participants provided written informed consent, prior to the experiment approved by the National Ethics Committee (CPP Sud Me´dite´ranne´e III, Nˆımes, France, #2009.07.03ter and ID-RCB-2009-A00513-54) conforming to the Declaration of Helsinki. The methods in the current study were carried out in accordance with the approved guidelines.

Exclusion criteria for both the clinical and nonclinical groups were (a) history of head trauma, (b) known neurological disease, (c) an actual ECT treatment, (c) substance abuse and or substance dependence (excluding tobacco and cannabis), and (d) people deprived of their liberty. All participants were native French speakers with a minimal reading level (validated using the fNART test) and were able to understand and perform the social-coordination task described in the following section.

**Details of Clinical Assessments**

All patients were interviewed by members of the specialized multidisciplinary team of the University Department of Adult Psychiatry, which belongs to a French national network of 10 Schizophrenia Expert Centers (Bordeaux, Clermont-Ferrand, Colombes, Créteil, Grenoble, Lyon, Marseille, Montpellier, Strasbourg, Versailles), set up by a French scientific cooperation foundation, FondaMental Foundation ([www.fondation-fondamental.org](http://www.fondation-fondamental.org/)) and created by the French Ministry of Research.

Except for the SCID, patients were assessed by trained clinical psychologists who rated the PANSS and other clinical scales after a unique clinical interview.

The clinical and cognitive assessments included the following evaluations:

- **Neurological Soft Signs Scale (NSS)** [[1](#_bookmark57)]
  Assesses subtle abnormalities in sensory-perceptual motor functions directly associated with schizophrenia pathology [[2](#_bookmark58), [3](#_bookmark59)] or induced by neuroleptic medications [[4](#_bookmark60)].
- **Positive and Negative Syndrome Scale (PANSS)** [[5](#_bookmark61)]

Ratings are made on a 7-point scale ranging from 1 (absent) to 7 (extreme). For this study, four of the analytically-derived PANSS factor component scores were taken into account: Total, General Psychopathology, Positive and Negative scores.

- **Trail Making Test (TMT, part A and B)**Part A requires the subject to use a pencil to connect 25 encircled number in numerical order. In part B, 25 encircled numbers and letters have to be connected by the subject in a numerical and alphabetical order. The letters and numbers are alternated to form a series such as A 1 B 2 C, etc. The performance is measured in terms of total time of completion for part A and B. It has also been proposed to calculate B-A difference between the completion time of these two tasks to minimize visuo-perceptual and working memory demands, providing a relatively pure indicator of executive control abilities [[6](#_bookmark62)]. In [[7](#_bookmark54)], authors reported a moderate to severe impairment on part A of the TMT in schizophrenia patients, and noted that impairments in processing speed can mask additional impairments that could be potentially observed in part B.
- **Mind Perception Questionnaire (MPQ)** [8]
  The MPQ was developed to evaluate how individuals perceive the mental capacities of various human and nonhuman characters. The MPQ evaluates how individuals perceive living and non-living things in terms of Experience (e.g. How much is the robot capable of experiencing physical or emotional pleasure?) and Agency (e.g. How much is the robot capable of remembering things?). In the version of the MPQ used in the iCub experiment, 5 questions were asked to the participants for each of the Agency and Experience components after the end of the trials.

**Humanoid Robot and Feedback Generation**

The right arm and the torso of the robot were used to perform human-like movements in the interactions with the participants. For this purpose, a standard inverse kinematics solver ensured an accurate tracking of the desired movements. Moreover, the mounted LEDs on the face of the robot (representing the mouth and eyebrows) were used to generate smiles as positive social feedback. For the nonsocial case a tablet was placed over the robot face displaying plus signs as positive nonsocial feedback. Finally, the neutral face of the robot (i.e., keeping all the LEDs off) was used for the neutral condition where no feedback was provided for the participants. The control parameters of the robot were fixed throughout the experiment, which allows for an unbiased evaluation of the effect of manipulated variables; i.e., nonsocial and social feedback.

In the feedback conditions, the robot provided the participants with positive feedback; i.e., smiles for the social and a plus sign for the nonsocial condition. A positive feedback was triggered if, compared to the last 5 seconds, the quality of the interaction was improved with respect to (1) the position error, (2) the velocity error, and (3) the sum of velocities. By having all three current factors below/above a certain percentile of their 5-seconds-history, the feedback was displayed for 1.5 seconds followed by a 3 seconds refractory period. Each trial received between 5 and 18 feedbacks (*M* = 11*.*5, *SD* = 1*.*8). A one-way Anova showed that schizophrenia patients received *.*36 less smiles (*F*(1*,* 657) = 6*.*43, *p* = *.*011). Moreover, another one-way Anova showed that each condition received statistically comparable numbers of feedbacks (*F*(2*,* 656) = *.*06, *p* = *.*939).

Even though no positive feedback was displayed to the participants in the neutral condition, we still computed the triggering of the feedback. This extra computation, which serves as a baseline in our statistical analysis, enabled us to study the bidirectional coupling between the participants’ synchrony and the frequency of the feedback. The participants’ synchrony triggers positive feedback for the robot, and the presence of positive feedback potentially influences the synchrony of the participants. In other words, we are interested to see how the correlation between the frequency of feedback and our measure of synchrony changes across conditions.

**Statistical Analysis**

The experimental design was composed of one independent between-subject variables, a group factor (control and schizophrenia group), and one independent within-subject variable, a condition factor (neutral, nonsocial, and social). The number of computed positive feedback during the interaction was considered as a covariate in the analysis. Demographic characteristics were statistically compared across groups using non-parametric U-Mann-Whitney test for continuous variables (e.g., age), and Chi-squared test for binary variables (e.g., gender). Pairwise comparisons between groups were performed using a t-test when necessary.

The time series of participants’ motions were recorded at 40*Hz* and low-pass filtered using a robust locally weighted polynomial regression with a span of 1%. To evaluate synchrony during the coordination task, we computed the average velocity error between the participants and the robot for each trial. This measure used as the dependent variable in our statistical analysis to study the effect of group and condition where we performed a multiple linear regression accompanied with the Analysis of Covariances (ANCOVA). For an easier interpretation of the result, we used a nested representation for the condition factor. A dummy variable (i.e., *Feedback*) compares the social and nonsocial conditions with the neutral condition; and a nested dummy variable (i.e., *Social*) compares the social to the nonsocial condition. This representation results in coefficients with 1 df; i.e., a set of simple pairwise comparison, and a clear relationship between linear regression and ANCOVA. Moreover, the group factor was coded as 1 for schizophrenia and +1 for control group. This leads to estimation of coefficients that are averaged across groups; see .

**Figure 1. The socio-motor coordination index (SMCi).**

This index is calculated based on the sensitivity of the synchrony index to the frequency of feedback; i.e., the estimated slope as shown on the figure. **(A)** The baseline slope (∆*B*) is extracted from the neutral condition. In this condition, the feedback is computed but not displayed to the participants. This baseline shows the sensitivity of the feedback-generation algorithm to the performance of the participants. **(B)** In a feedback condition, the causality is bidirectional. However, by subtracting the slope (∆*F* ) to the baseline, we focus only on the sensitivity of the participants’ synchrony to the number of feedback displayed by the robot. Here, the slope (∆*F* ) decreases compared to the baseline, showing a weaker coupling between synchrony and feedback compared to the neutral condition. This case illustrates an impeding effect of the feedback on synchrony. **(C)** Conversely, this case illustrates a facilitatory effect of the feedback on synchrony.

In human-human interaction, synchrony increases affiliation, and conversely, social cues indicating affiliation promotes synchrony. In our experiment, this causal loop between participants’ synchrony and robot’s feedback is also present. To account for this property, the frequency of the positive feedback was included in the model as a covariate. The estimated slope for this covariate shows how the synchrony index and the frequency of feedback are linearly related (or correlated); see Figure 1. In this bidirectional coupling between the two variables, we are interested in the sensitivity of the synchrony index to the frequency of positive feedback. To keep only this effect, the estimated slope in the neutral case (where feedback was computed based on the performance of the participants, but not shown) was used as a baseline; see Figure 1.A. The estimated slope in the feedback conditions (∆*F* ) is normalized to the estimated slope in the neutral condition (∆*B*) as follows.


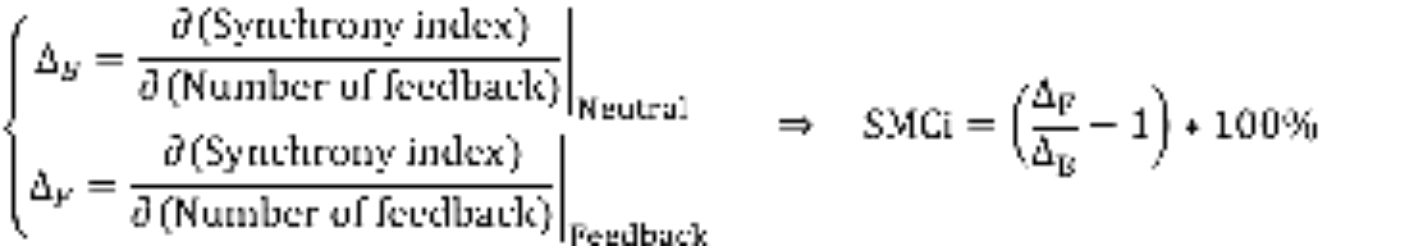


Negative values for *SMCi* signify a impeding effect (see Figure 1.B), whereas positive values signify a facilitatory effect for the feedback (see Figure 1.C).

For the linear regression, we considered a full factorial model except the inclusion of two interaction effects: (1) As the computed feedbacks in the neutral cases were not shown to the participants, the interaction effect between the covariate and group was excluded in this condition. This means one slope was extracted in the neutral condition, thus showing how the measure synchrony influences the frequency of the smiles. (2) The interaction effect between the covariate and group in the nonsocial condition was found to be insignificant and excluded from the model. This means that *SMCi* found to be statistically equivalent for both schizophrenia and control in the nonsocial condition.

In our analysis, the continuous variables are modified to make the effect and estimation of other variables interpretable. The average frequency of the feedback (11.5 feedback per trail) was removed from this variable. Moreover, we transformed our dependent variable using Box-Cox power transformation [9] (with *λ* = 1) to ensure the normality of the residual using Shapiro-Wilk normality test (*W* = 0*.*99 , *p* = 0*.*41). For further simplification, we linearly mapped this variable between 0 and 1; 0*/*1 indicating the worst/best performance among the participants. Finally, the equality of variances across groups and conditions were ensured using the Levene’s test, and no sign of heteroskedasticity was observed in the residuals.

For clinical correlation analysis, we used linear regression with the synchrony index as the dependent variable. In single predictor scenarios, the results are equivalent to the Pearson correlation test (i.e., same p-values with rho-squared equal to R-squared). To statistically compare the correlation coefficients, we used Fisher *z*-score with two-sides alternative hypothesis. We explored the correlation between medication dosage and synchrony with a Kendall Tau test.

**Analysis of the Mind Perception Questionnaire**

Comparing both groups, a U-Mann-Whitney test shows that patients attribute significantly higher agency to the robot than control participants (*W* = 152, *p* = .035). No significant effect of experience attribution was found.

To study the effect of agency attribution on motor coordination, a multiple linear regression was performed. This model predicts the synchrony index based on the group (i.e., control or patient) and the agency attribution score. A significant regression equation was found (*F*(3*,* 655) = 35.34, *p < .*001), with *R*^2^ = .13. This model shows that agency attribution has a statistically significant impeding effect on synchrony (*F*(1*,* 655) = 36.37, *p < .*001). However, no interaction effect between the group and the agency attribution score was found (*F*(1*,* 655) = .00, *p >.*9). Those results are presented in Table 2.

In terms of mind perception, the more schizophrenia patients and healthy controls perceived the humanoid robot as being capable of experiencing things and having a mind, the worse they performed in the task. In the same line, other studies have shown that a high attribution of agency (also evaluated with the MPQ) was negatively correlated to the frequency of use of an eldercare robot [10] Our results show that the effect of agency attribution on the performance in synchrony does not depend on the participants’ group (patients or controls), but is rather a general effect observed in the population.

***Table 1. Results of statistical analysis for synchrony and social motor coordination.***

Residual standard error: 0.1601 on 649 degrees of freedom. Multiple R-squared: 0.242, Adjusted R-squared: 0.2315. F-statistic: 23.02 on 9 and 649 DF, *p*-value *< .*001. Moreover, Levene’s test showed the equality of variance across groups (*F*(1*,* 657) = 2*.*67 , *p* = 0*.*102) and conditions (*F*(2*,* 656) = 1*.*05 , *p* = 0*.*35).

|  | ***Linear regression*** | | | | |  | ***ANCOVA*** | | | | |
| --- | --- | --- | --- | --- | --- | --- | --- | --- | --- | --- | --- |
| **Model** | **Estimate** | **Std. Error** | | **t-value** | **95% C.I.** |  | **SoS** | **df** | **F** | **Sig.** |  |
| Constant | 0.448 | 0.011 | 41.488 | | [ 0.427 , 0.469] |  | 44.128 | 1 | 1721.237 | 0.000 | *** |
| Goup | -0.054 | 0.011 | -5.006 | | [-0.076 , -0.033] |  | 0.642 | 1 | 25.058 | 0.000 | *** |
| Feedback | -0.003 | 0.015 | -0.196 | | [-0.033 , 0.027] |  | 0.001 | 1 | 0.039 | 0.844 |  |
| Feedback:Social | 0.010 | 0.015 | 0.674 | | [-0.020 , 0.040] |  | 0.012 | 1 | 0.454 | 0.501 |  |
| Feedback:Group | 0.001 | 0.015 | 0.079 | | [-0.029 , 0.031] |  | 0.000 | 1 | 0.006 | 0.937 |  |
| Feedback:Social:Group | 0.013 | 0.015 | 0.875 | | [-0.017 , 0.044] |  | 0.020 | 1 | 0.766 | 0.382 |  |
| N smiles | -0.046 | 0.006 | -7.382 | | [-0.058 , -0.033] |  | 1.397 | 1 | 54.494 | 0.000 | *** |
| N smiles:Feedback | 0.019 | 0.008 | 2.279 | | [ 0.003 , 0.036] |  | 0.133 | 1 | 5.196 | 0.023 | * |
| N smiles:Feedback:Social | -0.016 | 0.008 | -1.971 | | [-0.033 , 0.000] |  | 0.100 | 1 | 3.884 | 0.049 | * |
| N smiles:Feedback:Social:Group | -0.014 | 0.006 | -2.368 | | [-0.026 , -0.002] |  | 0.144 | 1 | 5.607 | 0.018 | * |
| Residuals |  | 0.160 |  | |  |  | 16.639 | 649 |  |  |  |

**Table 2. Results of statistical analysis for the Mind Perception Questionnaire**

Residual standard error: 0.1698 on 655 degrees of freedom. Multiple R-squared: 0.139, Adjusted R-squared: 0.1354. F-statistic: 35.34 on 3 and 655 DF, *p*-value *< .*001.

|  | ***Linear regression*** | | | |  | ***ANOVA*** | | | | |
| --- | --- | --- | --- | --- | --- | --- | --- | --- | --- | --- |
| **Model** | **Estimate** | **Std. Error** | **t-value** | **95% C.I.** |  | **SoS** | **df** | **F** | **Sig.** |  |
| Constant | -0.393 | 0.009 | -42.100 | [-0.412 , -0.375] |  | 51.125 | 1 | 1772.445 | 0.000 | *** |
| Group | -0.082 | 0.014 | -5.704 | [-0.11 , -0.054] |  | 0.939 | 1 | 32.538 | 0.000 | *** |
| Agency | -0.048 | 0.014 | -3.411 | [-0.076 , 0.020] |  | 0.336 | 1 | 11.634 | 0.000 | *** |
| Group:Agency | 0.001 | 0.017 | 0.088 | [-0.032 , 0.035] |  | 0.000 | 1 | 0.0078 | 0.910 |  |
| Residuals |  | 0.170 |  |  |  | 18.893 | 655 |  |  |  |

**References**

- 1. Krebs, M.-O., Gut-Fayand, A., Bourdel, M.-C., Dischamp, J. & Olie´, J.-P. Validation and factorial structure of a standardized neurological examination assessing neurological soft signs in schizophrenia. *Schizophrenia research* 45, 245–260 (2000).
  2. Gupta, S. *et al.* Neurological soft signs in neuroleptic-naive and neuroleptic-treated schizophrenic patients and in normal comparison subjects. *American Journal of Psychiatry* 152, 191–196 (1995).
  3. Walther, S. & Strik, W. Motor symptoms and schizophrenia. *Neuropsychobiology* 66, 77–92 (2012).
  4. D’Agati, E., Casarelli, L., Pitzianti, M. & Pasini, A. Neuroleptic treatments and overflow movements in schizophrenia: Are they independent? *Psychiatry research* 200, 970–976 (2012).
  5. Kay, S. R., Flszbein, A. & Opfer, L. A. The positive and negative syndrome scale (panss) for schizophrenia. *Schizophrenia* *bulletin* 13, 261 (1987).
  6. Sanchez-Cubillo, I. *et al.* Construct validity of the trail making test: role of task-switching, working memory, inhibi- tion/interference control, and visuomotor abilities. *Journal of the International Neuropsychological Society* 15, 438 (2009).
  7. Bowie, C. R. & Harvey, P. D. Administration and interpretation of the trail making test. *Nature protocols* 1, 2277–2281 (2006).
  8. Gray, H. M., Gray, K. & Wegner, D. M. Dimensions of mind perception. *Science* 315, 619–619 (2007).
  9. Yeo, I.-K. & Johnson, R. A. A new family of power transformations to improve normality or symmetry. *Biometrika* 87, 954–959 (2000).
  10. Stafford RQ, MacDonald BA, Jayawardena C, Wegner DM, Broadbent E. Does the robot have a mind? Mind perception and attitudes towards robots predict use of an eldercare robot. *International journal of social robotics*. 2014;6(1):17-32.
